# Supplementary material for: Different population dynamics in the supplementary motor area and motor cortex during reaching
Source: Nat Commun. 2018 Jul 16;9:2754. doi: 10.1038/s41467-018-05146-z (PMC6048147; doi:10.1038/s41467-018-05146-z)
Supplement: Supplementary file 1 — Supplementary figures [file 41467_2018_5146_MOESM1_ESM.pdf]

Supplementary information

Different population dynamics in the supplementary motor area and motor cortex during reaching

Lara et al.

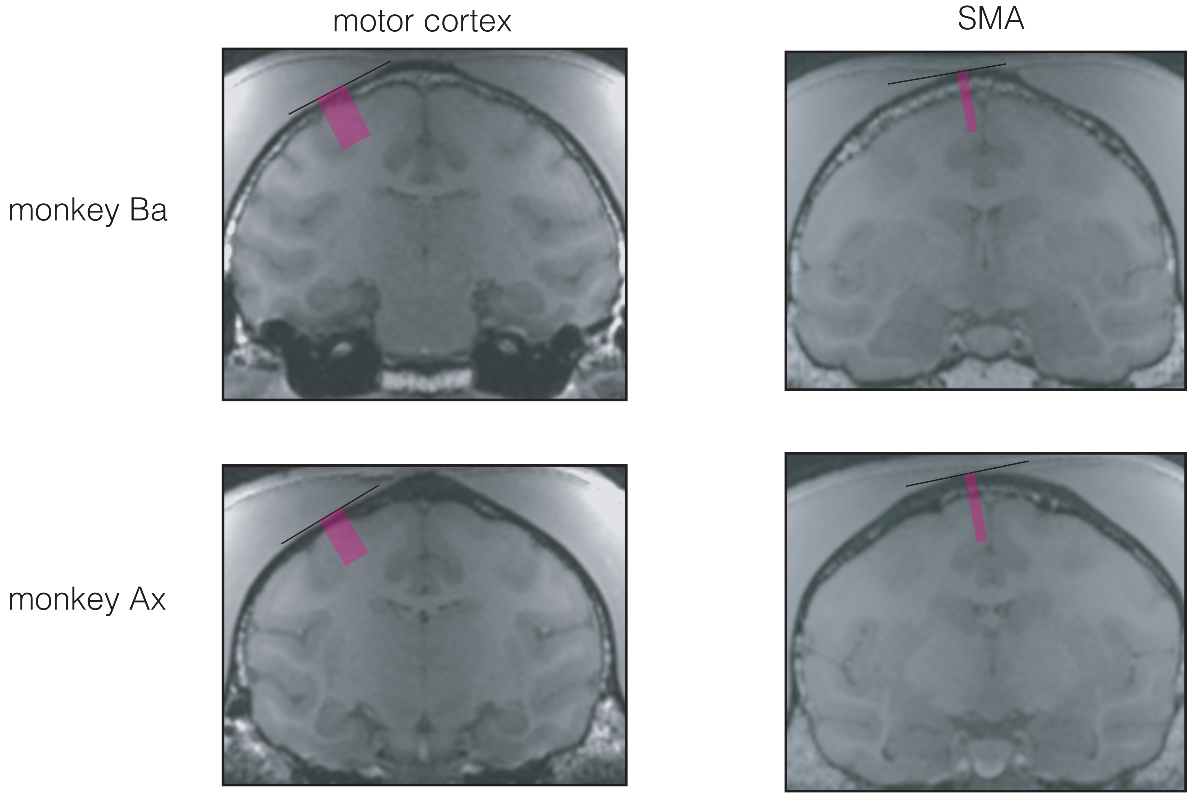

Supplementary Figure 1. Coronal MRI sections indicating cylinder placements (black line) and the approximate region containing recording locations (pink shading). Images are shown for a single coronal slice, but recordings spanned a considerable anterior-posterior range; see Figure 1 c,d. Microstimulation in the recorded regions of both motor cortex and SMA evoked movements of the arm – typically muscles of the upper arm and shoulder (Methods).

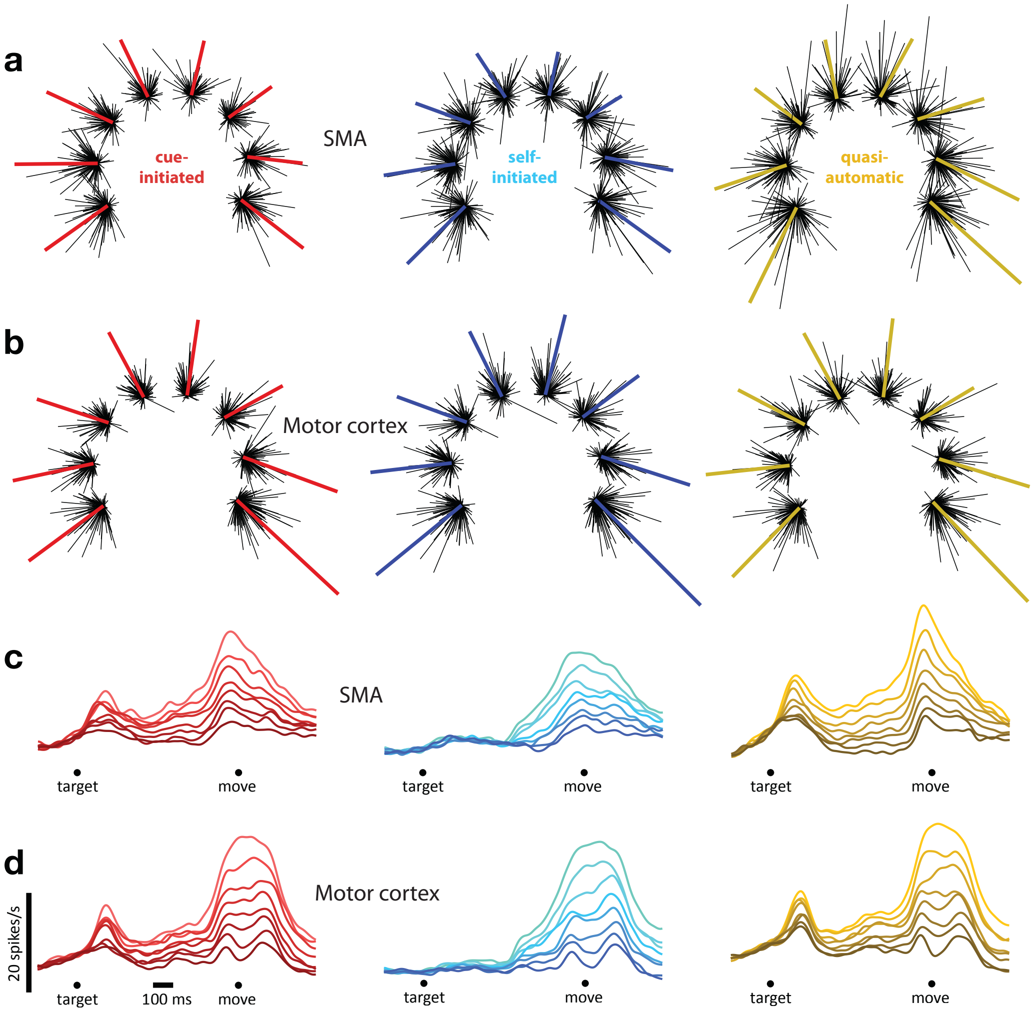

Supplementary Figure 2. Same as for Figure 3 but for monkey Ax. **a.** Population vectors for motor cortex. **b.** Population vectors for SMA. **c.** Population PSTHs for motor cortex. **d.** Population PSTHs. for SMA.

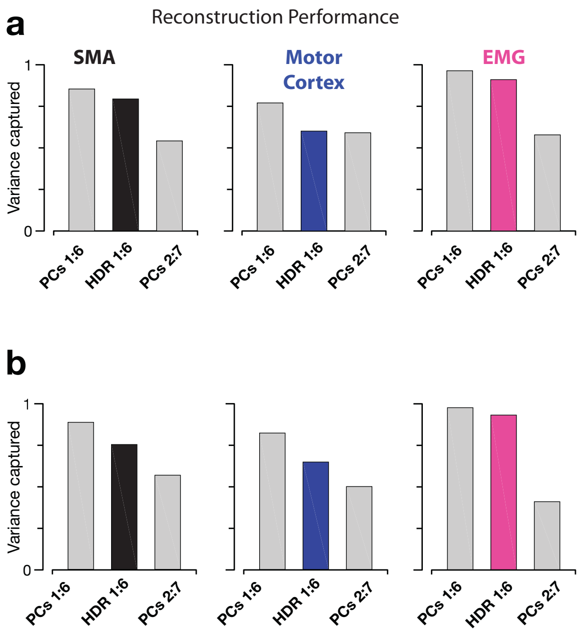

Supplementary Figure 3. Analysis of variance captured by HDR. **a.** Black bars plot the proportion of variance captured by the six dimensions identified via HDR. For comparison, gray bars plot the variance captured by the top six PCs, and by PCs two through seven. Variance captured reflects the accuracy with which individual neural responses can be reconstructed as weighted sums of the latent variables. Unity corresponds to all variance being captured and thus perfect reconstruction. Data are for monkey Ba. **b.** Same as A but for monkey Ax.
